# APOBEC Status Classification Using a Curated Gene Panel in Multiple Myeloma

**DOI:** 10.1002/hon.70269

**Published:** 2026-10-23

**Authors:** Dimitris Vraxnos, Paris Vogazianos, Cameron Brown, Marta Lionetti, Juan Carlos Entizne, Emily Charalambous, Athos Antoniades, Niccolo Bolli

**Affiliations:** ^1^ Stremble Ventures LTD Germasogeia Limassol Cyprus; ^2^ Department of Oncology and Hemato‐Oncology University of Milan Milan Italy; ^3^ Fondazione IRCCS Ca’ Granda Ospedale Maggiore Policlinico Milan Italy

**Keywords:** APOBEC, driver genes, multiple myeloma, mutational signatures, risk stratification, smoldering multiple myeloma

## Conflicts of Interest

The authors declare no conflicts of interest.

Multiple myeloma (MM) is typically preceded by precursor states such as monoclonal gammopathy of undetermined significance (MGUS) and smoldering multiple myeloma (SMM). While MGUS rarely progresses to active disease, patients with high‐risk SMM (HR‐SMM) face a substantially greater likelihood of developing symptomatic MM [1]. To address this, early therapeutic interventions for HR‐SMM are being evaluated in clinical trials, with several studies demonstrating increased progression‐free survival (PFS) and, in some cases, improved overall survival (OS) [2].

Despite these promising trial results, the routine treatment of HR‐SMM remains debated due to imperfect risk stratification. Current SMM risk scores [3] primarily rely on surrogates of disease burden rather than true biological drivers of aggressiveness, which risks misclassification and subsequent over‐ or under‐treatment. SMM is highly heterogeneous; despite similar disease burdens, some patients maintain an indolent, MGUS‐like profile, while others harbor genetic signatures mirroring active MM long before clinical progression occurs [4]. Consequently, there is a critical need for reliable prognostic tools that capture the underlying biology of these precursor stages.

Mutational signatures, particularly those driven by the APOBEC family of cytidine deaminases, are among the most robust genomic predictors of progression and signify high‐risk disease in both SMM and active MM [4, 5]. While clinical next‐generation sequencing (NGS) routinely relies on targeted panels to detect specific mutations and structural variants [6], identifying APOBEC signatures has traditionally required whole‐exome (WES) or whole‐genome sequencing (WGS), limiting its routine clinical utility [7]. To address this, we investigated whether a curated gene panel, analyzed via a direct mutation‐counting approach, could serve as a practical clinical surrogate.

To assess the feasibility of this approach, a dataset from Multiple Myeloma Research Foundation (MMRF) CoMMpass study (IA21 [8]) was utilized. Pre‐annotated Variant Call Format (VCF) file from 1156 patient samples with available WES data was analyzed. As a “gold standard' classification was established using the mmsig *R* package [9] to classify patients based on the full exome data: 764 patients were identified as APOBEC‐positive, defined by the presence of single base substitution signatures SBS2 and/or SBS13 (activity > 0) and 392 as APOBEC‐negative.

This was compared to a panel‐based approach using a deterministic “direct count' method to mimic clinical workflows. After assembling a list of 246 known myeloma driver genes, the algorithm filtered for variants exclusively within their coding regions. Then identified single base substitutions characteristic of APOBEC activity specifically Cytosine to Thymine (*C* > *T*), Cytosine to Guanine (*C* > *G*), or Cytosine to Adenine (*C* > *A*) and verified their sequence context using the provided annotations. Only mutations occurring within a TpC dinucleotide context (where a thymine precedes the mutated cytosine 5′‐T‐C‐3′) were counted, as this is the preferred substrate for APOBEC enzymes. Patients were classified as APOBEC positive if the total number of these qualifying mutations across the panel was two or more.

Concordance and performance analysis showed: Targeted panel correctly identified 507 of the 764 APOBEC‐positive patients and 227 of the 392 APOBEC‐negative patients yielding an overall Accuracy of 63.5%, Sensitivity of 66.4% and Specificity of 57.9%. In terms of clinical utility, the Positive Predictive Value (PPV) was 75.4% with a Positive Likelihood Ratio of 1.58. Although the sensitivity indicates that some patients with low mutational burden were not detected, the relatively high PPV suggests that a positive result on the panel is a strong indicator of APOBEC activity.

To assess whether the targeted panel retained prognostic value despite reduced sensitivity, we conducted overall survival (OS) analyses (Figure 1). Both the gold‐standard WES approach and our targeted panel successfully stratified patients (*p* = 0.005 and *p* = 0.012, respectively), reaffirming the adverse prognostic impact of APOBEC activity [5].

**FIGURE 1:**
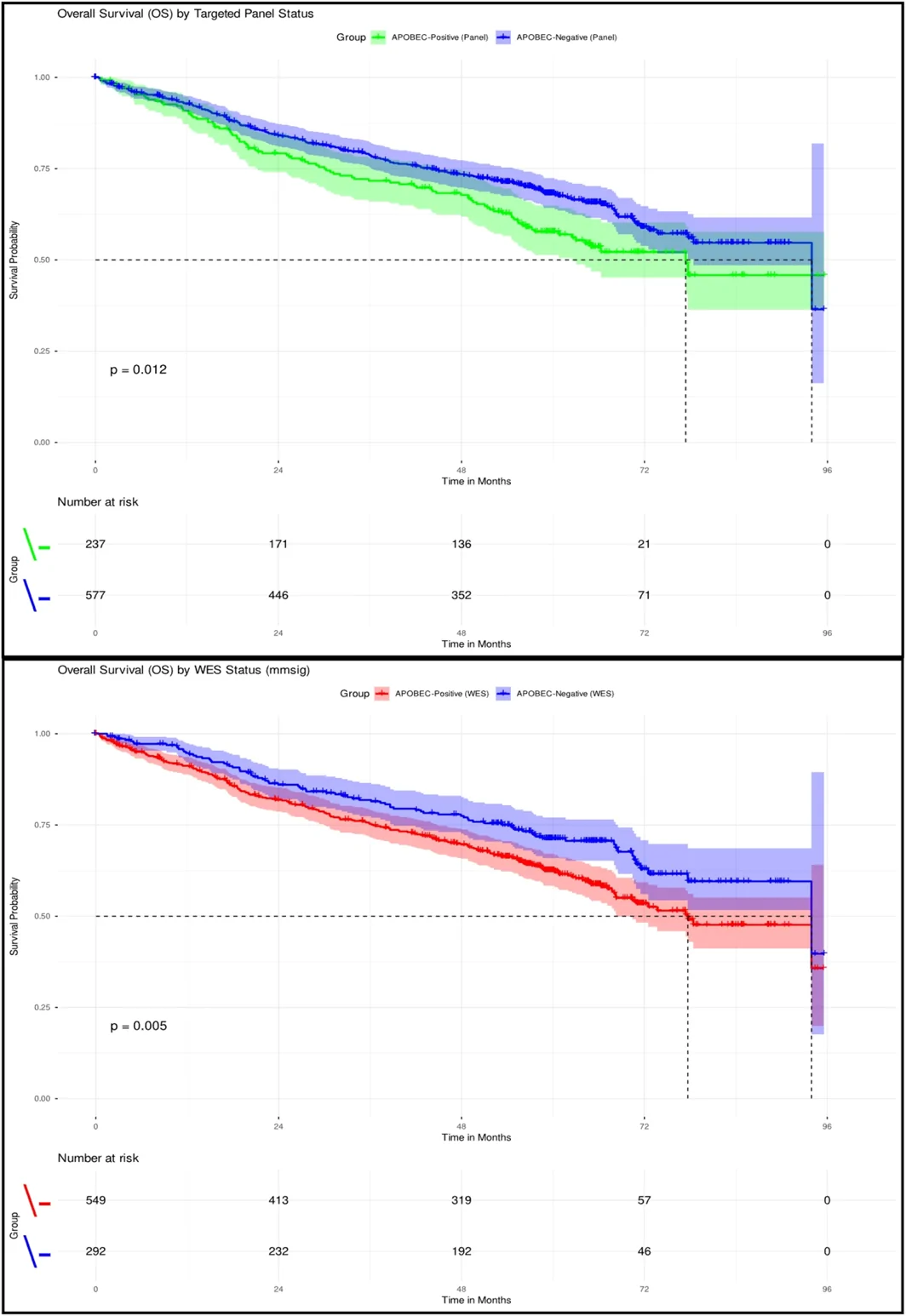
Kaplan–Meier analysis of overall survival (OS) stratified by APOBEC status. (A) The Targeted Panel achieves significant separation between APOBEC‐positive and APOBEC‐negative patients (*p* = 0.012). (B) The gold‐standard whole‐exome sequencing (WES) approach yields significant separation (*p* = 0.005).

Whether gene selection strategy influences performance was further explored via simulation to estimate the minimum number of genes required to ensure robustness (Figure 2). To this end, a bootstrapping simulation was designed defining a range of panel sizes (10, 60, 120, 180, 240, 500, and 1000 genes). For each size, 30 iterations of both a ‘Targeted’ panel (drawing from the curated list) and a ‘Random' panel (drawing from the whole exome) were generated. For each simulated panel, classification accuracy was assessed using the maximum value of Youden's Index to account for the trade‐off between sensitivity and specificity.

**FIGURE 2:**
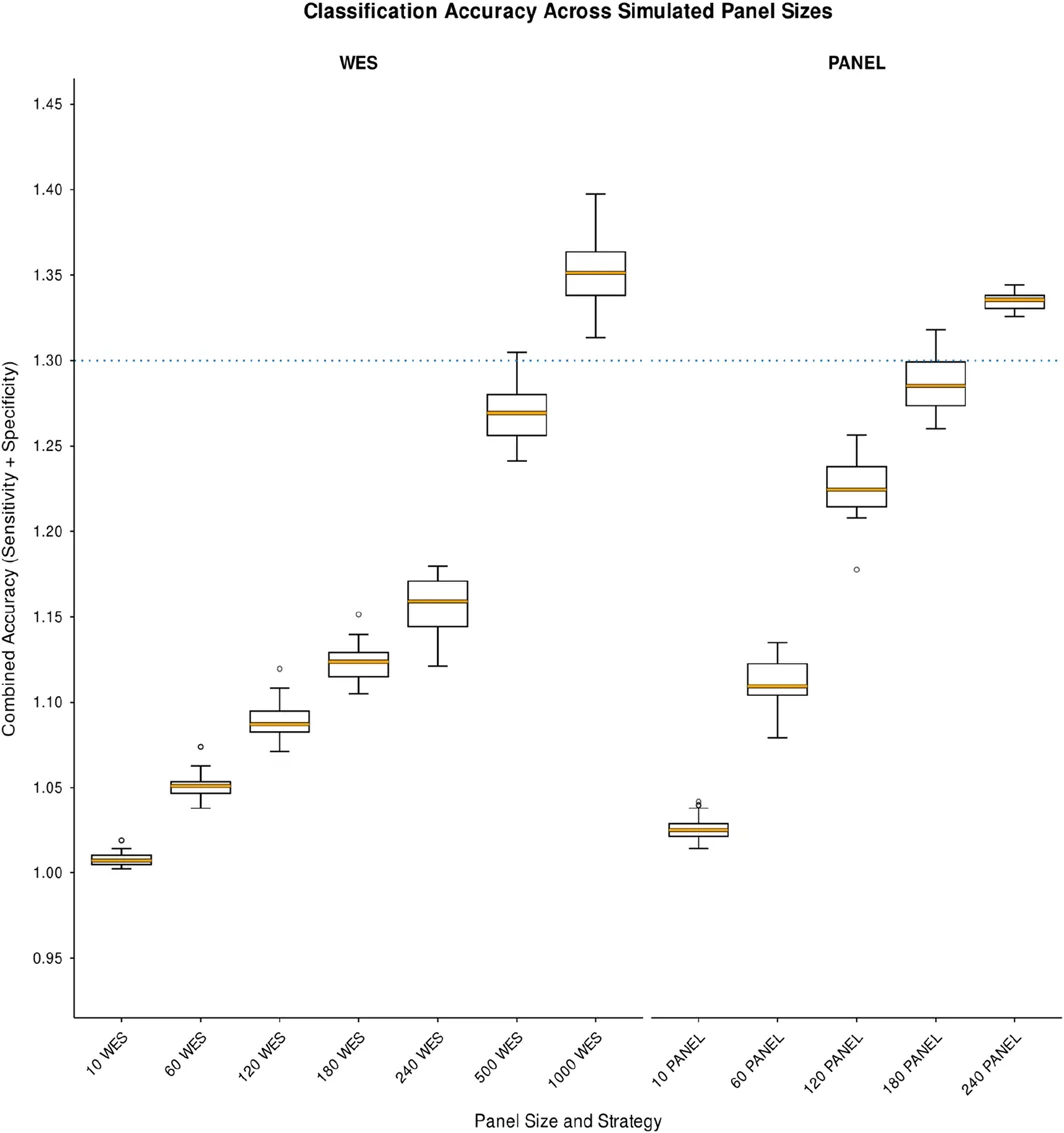
Simulation analysis comparing classification performance of randomly selected genes (WES) versus the rationally designed targeted panel (PANEL). Boxplots represent the distribution of combined accuracy (Sensitivity + Specificity) across 30 iterations for each panel size (10–1000 genes). The blue dotted line represents the benchmark for clinical utility (Youden's *J* > 0.3, equivalent to *y* > 1.3). The targeted panel achieves this threshold with approximately 180 genes, whereas random selection requires significantly larger panels (∼500 genes) to reach comparable performance.

The results suggest that a rationally designed panel may offer a feasible approach: Across all panel sizes, the median accuracy of the ‘Targeted' panels exceeded that of ‘Random' panels. The Youden's *J* threshold of > 0.3 was set as a benchmark for clinical utility. Notably, a targeted panel comprising of 180‐gene consistently met this threshold, whereas randomly selected panels required approximately 500 genes to achieve similar performance.

Since APOBEC‐induced mutations are scattered randomly across the genome but preferentially cluster in areas of hypermutation called “kataegis” (which are mostly intergenic), it is important to consider why a panel curated around myeloma genes would perform better than a random one. Indeed, a curated panel of myeloma driver genes offers a higher chance of detecting an increased number of mutations than a randomly selected one, and reinforces the notion that APOBEC is implicated in determining myeloma aggressiveness through mutations of driver myeloma genes [5].

Although assessing APOBEC activity in SMM and newly diagnosed multiple myeloma (NDMM) has significant prognostic value [10] current methods rely on comprehensive genomic profiling, which limits their widespread clinical adoption [7]. The sensitivity (66.4%) and specificity (57.9%) observed in this study should be considered in light of clinical accessibility: a diagnostic tool that is sufficiently accurate and widely deployable may offer greater population level benefit than a perfect but impractical test [7]. Limitations of this study include its retrospective design and reliance on a computational comparator; prospective validation in independent datasets will be required.

In conclusion, our findings support the feasibility of a cost‐effective, targeted sequencing assay. A rationally designed panel of just 180 genes achieved moderate classification accuracy and retained the statistical power necessary to stratify high‐risk patients. Pending validation, this tool could broaden clinical access to APOBEC status as a prognostic biomarker, improving risk stratification in both precursor stages and at the time of active disease diagnosis.

## Author Contributions

D.V. and A.A. designed the study. N.B. collected the data. D.V. and P.V. analyzed the data. D.V. wrote the paper. N.B., C.B. and E.C. review and editing. All authors participated in result discussions, critically reviewed the manuscript, and approved the final version for publication.

## Funding

This work was funded by the European Union (ERC Proof of Concept, Myeloma Risk, 101123230).

## Data Availability

The data that support the findings of this study are openly available in MMRF Researcher Gateway at https://mmrf.formstack.com/forms/mmrf_virtual_lab_access_request.
